# Coupling and Coordination Relationship between the Tourism Economy and Ecosystem Service Value in Southern Jiangsu, China

**DOI:** 10.3390/ijerph192316136

**Published:** 2022-12-02

**Authors:** Bin Wang, Chunguang Hu, Jianxiong Li

**Affiliations:** 1 School of Social Science, Soochow University, Suzhou 215123, China; 2 School of Architecture and Urban Planning, Huazhong University of Science and Technology, Wuhan 430074, China; 3 Hubei Engineering and Technology Research Center of Urbanization, Wuhan 430074, China; 4 Independent Researcher, Vojvode Stepe Stepanovića 1, Stan 12, Kaluđerica, 11130 Grocka, Serbia

**Keywords:** ecosystem service value, tourism economy, coupling coordination model, southern Jiangsu

## Abstract

The relationship between the tourism economy and the ecosystem service value (ESV) is crucial for sustainable regional development. This study takes southern Jiangsu as a research object. Firstly, the development level of the tourism economy and ecosystem service value in southern Jiangsu from 2000 to 2020 are evaluated with the entropy method, ecosystem service value is estimated and the dynamic degree of land use is computed. Secondly, the coupling coordination degree model is used to explore the coupling coordination degree between the two systems. Finally, the interaction mechanism between the tourism economy and ecosystem service function is elaborated. The result shows that: (1) There are disparities in the levels of a comprehensive tourism economy in different cities, and the overall development level of the tourism economy in southern Jiangsu shows a cyclical fluctuation pattern. (2) Spatial variation of ecosystem service value exists in different cities in southern Jiangsu, with an overall trend of increasing in the beginning followed by a decline. (3) The coupling coordination degree between the tourism economic system and ecosystem service functions in southern Jiangsu demonstrates an inverted U-shaped development pattern from 2000 to 2020, evolving from mild disorder to intermediate coordination and then back to mild disorder, and the development of two subsystems is unstable and imbalanced. Within the region, Nanjing, Suzhou and Zhenjiang have experienced a rise in coupling coordination degree followed by a decline. This study also reveals the coupling mechanism between ecological service functions and the tourism economic system, and provides suggestions for ecological preservation and sustainable development of tourism industry in southern Jiangsu. This research can be a reference for tourism and regional development in southern Jiangsu and the whole Yangtze Delta region.

## 1. Introduction

The 2030 Agenda for Sustainable Development is one goal that the UN has set to be attained through green, inclusive and sustainable tourism [1,2]. With the increasing impact of tourism development on the ecological environment, the issue of coupled and coordinated development of the regional tourism economy and ecological environment has become a key issue in current governmental decision-making and academic research [3,4,5,6]. Whether the tourism economy and ecological environment are coordinated is an important criterion to assess the healthy and sustainable development of regional tourism [7,8], which can be observed according to changes in the degree of coordination [9]. The interaction mechanism between the tourism industry and the ecosystem services and functions is also a key foundation for academic study and policy-making [10,11]; thus, providing a sound scientific basis for the steady growth of the local natural ecosystem and the socioeconomic system is essential [12].

Research on both the tourism economy and ecosystem services started a long time ago. First, the concept of tourism economy was introduced at the beginning of modern tourism in the 19th century [13]. Some scholars have already noticed the huge economic impact of modern tourism and, thus, took the tourism economy as a new research direction and gradually formed a framework [14,15]. A tourism economy involves an active market, increased foreign currency income and increased employment opportunities, so it promotes regional economic development [16,17]. The development of a tourism economy is mainly reflected by the improvement of tourism facilities, the expansion of tourism market scale, the growth of profit from the tourism industry, and the increased number of workers in the tourism industry [18,19,20]. Second, the concept of ecosystem services has gradually emerged with the in-depth study of ecosystem structure, function and processes [11,21]. Ecosystem services refer to all the benefits directly or indirectly obtained by human beings from the ecosystem to meet the needs of human life and social development [22,23]. Ecosystem service functions are classified into the categories of regulation service, support service, supply service and cultural service [24,25]. Supply services are the supply of food, raw materials and water resources by the ecosystem [26]. Regulation services refer to the ecosystem function of gas regulation, climate regulation, environment purification and hydrological regulation [27]. Support services refer to soil conservation, maintenance of nutrient cycling and biodiversity [28]. Cultural services refer to the function of providing leisure, entertainment and aesthetic enjoyment for people [29]. The ecosystem service function of different types of land use can provide the fundamentals for tourism economic development [30,31].

**Figure 1:**
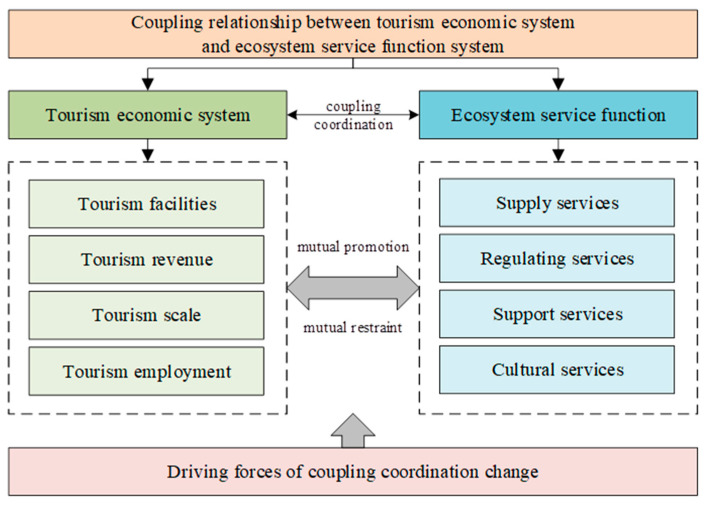
Coupling relationship between a tourism economic system and ecosystem service function system.

Figure 1 depicts the mutual promotion and mutual restraint existing between the tourism economic system and ecological service function [32]. The quick growth of the tourism industry can affect the ecosystem services [33], while ecosystem services also act on the efficiency and future direction of the tourism industry [34]. Meanwhile, there are interactions among factors within both systems. The development of a tourism economy should be based on urban ecological environment, and the ecological environment can also promote or hinder the development of a tourism economy [35,36]. The diversification of land-use types, such as water area, woodland and grassland, can provide ecological space for regional tourism development—thus attracting more tourists to come for leisure activities—and facilitate tourism economic growth [5,9]. However, the construction of tourism infrastructure results in a transition of land use, such as the transition from forests or agricultural land to urban land, and such transition degrades ecosystem service and decreases its value [10,35]. In addition, the growing number of tourists and the construction of human infrastructure can obviously affect the local ecosystem [4,8]. Therefore, it is necessary to explore the interaction mechanism between a tourism economic system and ecosystem service functions.

The literature review on the coupling relationship between tourism economy and ecosystem includes research methodologies, research data and research objectives. (1) In terms of research methodologies, the coupling coordination degree can represent the extent of interaction among several complex systems [37]. In some studies, researchers found a positive coupling on tourism economy and ecological environment after analysis, which means these two indices positively interact with each other and develop in a harmonized manner [38,39]. Some researchers built a comprehensive evaluation function or coupling model to quantitatively analyze the coupling coordination degree between a tourism economy and ecological environment, and observed that these two indices are highly associated [4,40]. (2) In terms of research data, most existing studies on the coupling coordination of tourism economies and ecological environment factors established indicator systems based on the data from statistical yearbooks [41]; however, it is not common to evaluate the relationship between the two indices with land-use data and ecological service value. The effective monitoring and management of regional land use can improve regional planning and provide fundaments for calculating ecosystem service value (ESV) [42]. (3) In terms of research objectives, the interaction mechanism between ecological services and an increase in the tourism economy has not been fully explored [43]; therefore, it is meaningful to explore this mechanism.

Due to the deficiency of relevant studies on the interaction between ecological service function and tourism economic growth, this study uses southern Jiangsu as an example, since this region has undergone rapid growth of its tourism economy. The comprehensive development level of a tourism economy and the extent of ecosystem service value (ESV) change in southern Jiangsu from 2000 to 2020 will be calculated through the entropy method; the comprehensive development index, the ESV and the land-use transition will also be explored. Then, the coupling coordination degree of tourism economy and ecosystem service functions in 2000, 2010 and 2020 is verified by a coupling coordination model. Finally, the coupling mechanism between tourism economic systems and ecosystem service function systems is analyzed. This study enriches the research on the coordinative relationship between the tourism economy and ecosystem service function, and provides a theoretical base for the protection of ecological environments and the optimization of tourism development in southern Jiangsu.

## 2. Materials and Methods

### 2.1. Study Area

**Figure 2:**
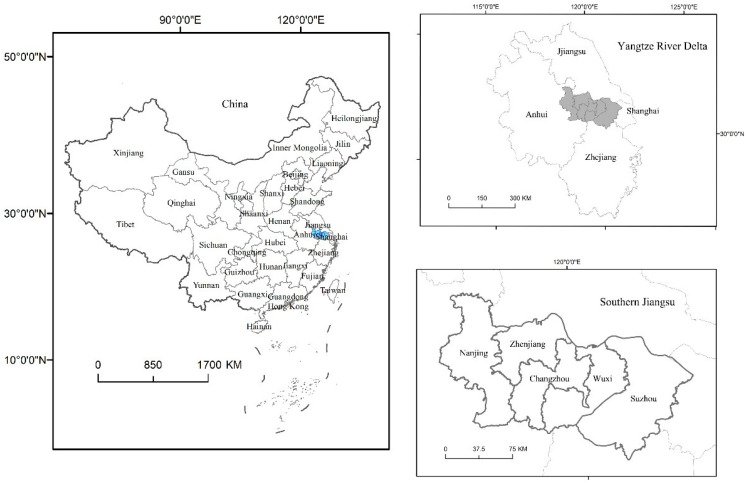
Study area.

As shown in Figure 2, southern Jiangsu is one of the most developed regions in China in terms of its tourism industry, as well as one of the most economically developed regions in Jiangsu Province [44]. Southern Jiangsu is the region that lies between 118.35° E and 121.38° E and 30.759° N and 32.611° N. The area covers a total area of 28,084 square kilometers and includes five prefecture cities, Nanjing, Suzhou, Wuxi, Changzhou and Zhenjiang. The population is 38.0239 million. In 2020, the total GDP in southern Jiangsu was CNY 5938.429 billion, with a per capita GDP of almost CNY 156,000, reaching the level of developed countries. The country’s urbanization rate has reached 70%. In southern Jiangsu, there will be 17 excellent tourist cities by 2022, making up the 61% of the provincial total, as well as 14 National AAAAA-Level Scenic Spots which make up 82% of Jiangsu’s total. Southern Jiangsu is one of the regions in China with abundant tourism resources; it is also an important hub for tourists throughout the whole province, and even the whole of China [45]. The value of ecosystem services has been impacted by the quick development of tourism in southern Jiangsu. Understanding the connection between regional tourism economic development and changes in ecosystem service value in this region is critical for promoting sustainable regional development, as this region is undergoing a rapid economic growth that is representative of China.

### 2.2. Data Source

The data for this study involves socioeconomic statistics and land-use data. The Resource and Environmental Science Data Center of the Chinese Academy of Sciences provided 2000, 2010 and 2020 land-use data of southern Jiangsu with 30 m resolution. The land use was classified into categories of cultivated land, forest land, grassland, water area, urban built-up land and unused land in ArcGIS; the data on the tourism industry, population data, food data and other socioeconomic data are extracted from the Jiangsu Statistical Yearbook, China Statistical Yearbook and the Statistical Communiqué of each city in Southern Jiangsu on economic and social development.

It is common to use grid cells in GIS as the carriers of indices, and a grid cell can be the unit of analysis and evaluation as well. The land-use data of 2000, 2010 and 2020 was converted from raster to vector using ArcGIS, and the ecosystem service value of each grid cell was computed. This technique overcomes the restriction of administrative borders through the reconstruction of land-use data at the grid scale.

### 2.3. Construction of the Index System of Tourism Economic Systems

#### 2.3.1. Selection of Indicators

It is necessary to construct a scientific index system to evaluate the coupling coordination between tourism economic systems and ecosystem service function. The index system is constructed according to relevant studies [7,45,46] and the current development of tourism in southern Jiangsu. Considering the representativeness, systematization and accessibility of indices, 9 indicators are selected to reflect the tourism development level. These 9 indicators can be sorted into four dimensions including tourism facilities, tourism revenue, tourism scale and tourism employment (Table 1). The tourism facilities dimension is measured by the number of scenic spots, the number of travel agencies and the number of star-grade hotels, which can represent the attractiveness and service capacity of the tourist destination. The tourism revenue dimension is measured by income from domestic tourism and foreign currency income from tourism, and the income is converted from USD to RMB with the exchange rate of the respective years. The tourism scale dimension is measured by the number of domestic tourists and the number of overseas tourists, which directly reflect the scale of the local tourism market. The tourism employment dimension is measured by the number of workers in the accommodation and catering industry, as well as the number of workers in the culture, sports and entertainment industry, which represent the social benefits of tourism development. Similarly, the index system of ecosystem service function includes 11 indices in four dimensions: regulation service, support service, supply service and cultural service.

#### 2.3.2. Determination of Index Weight

In order to objectively evaluate the development level of the tourism economy in southern Jiangsu, it is necessary to weigh the evaluation indicators. This paper uses the entropy weight method which is a commonly used objective weighting method in ecological environment studies for the comprehensive evaluation of multiple indicators [47]. The entropy weight method determines the indicator weight according to the variation degree of the value of each indicator [48]. Firstly, the indices are normalized by the min–max normalization method. Then, the weights of indicators at all levels are determined. Finally, the score of the tourism economic development of each city is computed. The method is as follows:

(1) $$\bar{x_{ij}}=\frac{x_{ij}-x_{jmin}}{x_{jmax}-x_{jmin}}$$

(2) $$\bar{x_{ij}}=\frac{x_{jmax}-x_{ij}}{x_{jmax}-x_{jmin}}$$

where $\bar{x_{ij}}$ is the normalization of $x_{ij}$, $x_{ij}$ is the original value of the *j*-th indicator of the *i*-th object, $x_{jmax}$ is the maximum value of the *j*-th index and $x_{jmin}$ is the minimum value of the *j*-th index. Formula (1) is for positive indicator normalization, while Formula (2) is for negative indicator normalization.

After obtaining the normalized indices, the entropy value of each index is calculated using the entropy weight method. The formula is as follows:

(3) $$e_{j}=-\frac{1}{\ln m}\overset{n}{\underset{j=1}{\sum }}p_{ij}\ln p_{ij}$$

(4) $$p_{ij}=\frac{\bar{x_{ij}}+1}{\sum_{i=1}^{n}\left(x_{ij}+1\right)}$$

(5) $$\omega_{ij}=\frac{1-e_{j}}{\sum_{j=1}^{m}\left(1-e_{j}\right)}$$

where *e_j_* is the entropy of the *j*-th index, *p_ij_* is the proportion of the *i*-th object in the *j*-th index, $\omega_{ij}$ is the weight value of the *j*-th index, *m* is the number of objects and *n* is the number of indicators.

Linear weighting is used to comprehensively evaluate the tourism economic development of each city. The formula is as follows:

(6) $$S\left(x_{i}\right)=\overset{m}{\underset{j=1}{\sum }}{\bar{x}}_{ij}\omega_{j}$$

where $S\left(x_{i}\right)$ is the tourism economic development index of the *i*-th object, which represents the capacity of tourism economic development. Similarly, the level of ecosystem service value is also calculated by the entropy method.

### 2.4. Dynamic Degree of Land-Use Change

Dynamic degree of land-use change is the degree to which the same type of land use changes through time [49]. The degree of dynamic land use can describe the quantity, types and the extent of the changes to land use over a certain amount of time [50]. The area variation rate of a certain land-use type over a specific period of time in a region can be measured by the dynamic degree of change in a land-use type [51]. The formula is as follows:

(7) $$L=\frac{M_{b}-M_{a}}{M_{a}}\times \frac{1}{F}\times 100\%$$

where *L* is the dynamic degree of change in a certain land-use type in the region, $M_{a}$ and $M_{b}$ are the areas of a certain land-use type at the beginning and end of the study and F is the length of time.

### 2.5. Estimation of Ecosystem Service Value

The value-transformation of ecosystem services is the core of coordination assessment. Ecosystem service value (ESV) quantitatively reflects the ecosystem service function of a region [52]. The concept of ESV was introduced by Constanz et al., and then improved by Xie Gaodi who established an ESV equivalent factor table based on the social reality of China [53]. This study established the ESV equivalent factor table of southern Jiangsu, as shown in Table A1 (Appendix A). The formula for calculating ESV equivalence factor is as follows:

(8) $$\;VC_{k}=\frac{1}{7}\times P\times \frac{1}{n}\sum_{i=1}^{n}Q_{i}$$

where $VC_{k}$ is the value of ESV equivalent factor (yuan · hm^−2^ · a^−1^); P is the national average grain price (yuan · kg^−1^); $Q_{i}$ is the average grain yield in the study area (kg · hm^−2^); and *n* is the number of years. The calculation of ecosystem service value is as follows:

(9) $$ESV=\sum \left(A_{k}\times VC_{k}\right)$$

where *ESV* is the ecosystem service value; $A_{k}$ is the area of type *k* land use (hm^2^); and $VC_{k}$ is the value of ESV equivalent factor (yuan · hm^−2^ · a^−1^).

### 2.6. Coupling Coordination Model

The coupling coordination model builds a tourism economy system and ecosystem service function, and then assesses the interaction and coordination between two systems [54]. The model is as follows:

(10) $$T=\alpha U_{1}+\beta U_{2}$$

(11) $$C=\frac{2\sqrt{U_{1}U_{2}}}{U_{1}+U_{2}}$$

(12) $$D=\sqrt{CT}$$

where *D* is the coupling coordination degree, *C* is the coupling degree, *T* is the comprehensive coordination index between systems, *U*_1_ is the comprehensive evaluation value of the tourism economy system and *U*_2_ is the comprehensive evaluation value of the ecological environment system. The tourism economy development level and the ecosystem service value level are measured based on the entropy weight method, respectively. *α* and *β* are equally assigned 0.5. The closer the *C* value is to 1, the stronger the interaction that exists between the two systems. However, the coupling model can only reflect the degree of influence between the two systems [5]. Therefore, based on the coupling model, the coupling coordination model is introduced and calculated by formula. According to relevant studies, the coupling coordination degree is classified as shown in Table A2 (Appendix A) [38,55]. In view of the characteristics of the research object in this paper, the coupling coordination degree is classified into 10 grades.

## 3. Results

### 3.1. Analysis of the Change in Comprehensive Development Level of Tourism Economy

**Figure 3:**
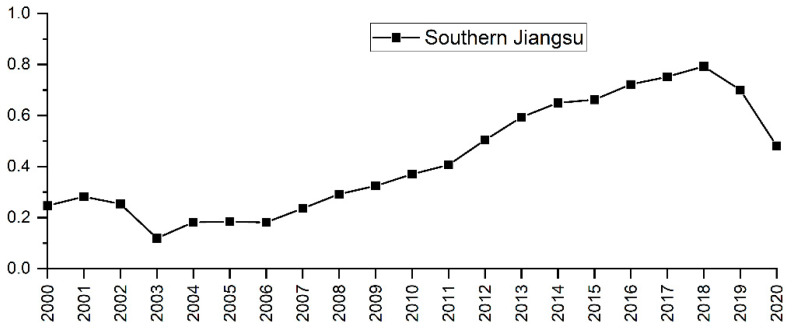
Overall tourism economic development level in southern Jiangsu.

The degree of the tourism industry’s comprehensive development in southern Jiangsu from 2000 to 2020 is determined using the entropy weight method. As can be seen in Figure 3, regarding the temporal dimension, the development level of the tourism economy in southern Jiangsu shows a cyclical fluctuation from 2000 to 2020. In particular: (1) From 2000 to 2010, the development level of the tourism economy in southern Jiangsu was at a primary development stage. The comprehensive development index of the tourism economy was 0.250 in 2000 and 0.370 in 2010, showing an increase of 0.120. (2) From 2010 to 2020, the development level of the tourism economy in southern Jiangsu was in a stage of stable rise followed by a sudden decline. In 2018, the comprehensive development index peaked at 0.793, and then began a downward trend due to COVID-19.

**Figure 4:**
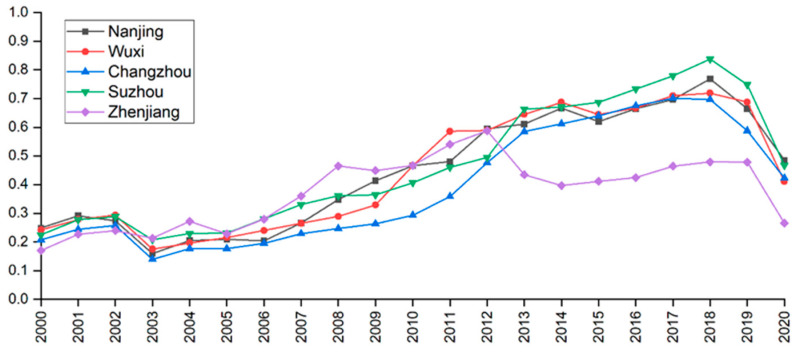
Comprehensive development level of the tourism economy of five cities in southern Jiangsu from 2000 to 2020.

There are some variations in the tourism economy development among the five cities in southern Jiangsu including Nanjing, Wuxi, Changzhou, Suzhou and Zhenjiang. Figure 4 shows that the tourism economic development levels of the five cities were comparable before 2012, whereas after 2014, Suzhou gradually rose to the top in southern Jiangsu; Wuxi, Nanjing and Changzhou had similar tourism economic development levels, and Zhenjiang had the lowest.

### 3.2. Temporal and Spatial Differentiation Characteristics of Ecosystem Service Value

#### 3.2.1. Spatio-Temporal Characteristics of Land-Use Change

1. Analysis of current land use

According to the interpretation of remote sensing images and the calculation of land use’s degree of dynamic change, the area of agricultural land decreased significantly—while the area of built-up land increased significantly—and the area of other land-use types remained stable in southern Jiangsu from 2000 to 2020. Table 2 shows that the primary land use is agricultural land, followed by water bodies and built-up land. Unused land makes up the smallest proportion. The area of agricultural land shrank from 2000 to 2020, and its dynamic change was −1.153%. Built-up land substantially expanded. In 2020, there was 662,639.6 hm^2^ of built-up land in southern Jiangsu, showing an increase of 7.280% compared to 2000. The regional development in southern Jiangsu is imbalanced, since new built-up land is mostly in urbanized areas.

2. Analysis of Land-Use Conversion

**Figure 5:**
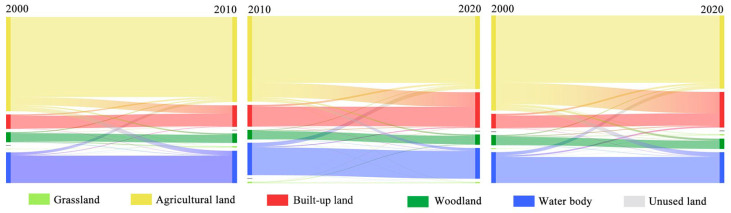
Land-use conversion map of southern Jiangsu from 2000 to 2020.

Table 3 and Figure 5 show the transition between each land-use type from 2000 to 2020. Agricultural land underwent the most outgoing conversion, primarily to built-up land. Water bodies underwent the second most outgoing conversion, primarily to agricultural land. Built-up land had the most incoming conversion from 2000 to 2020, mostly from agricultural land followed by water bodies and woodland, while the conversion from other types of land to built-up land is not relatively obvious. Such land-use conversion indicates that southern Jiangsu went through rapid urbanization from 2000 to 2020.

#### 3.2.2. Spatial–Temporal Differentiation of Total Land Ecosystem Services Value

Based on the revised coefficient of ecosystem service value per unit area (Table A1, Appendix A) and land-use data, the ESVs of southern Jiangsu from 2000 to 2020 were estimated (Table 4). The result shows that the ecosystem service value in 2000, 2010 and 2020 was about CNY 833.367 billion, CNY 862.675 billion and CNY 816.411 billion, respectively, showing a downward fluctuation. The ESVs of different land-use types are ranked as follows: water body > agricultural land > woodland > grassland > unused land. The ESV of agricultural land showed a trend of declining; the ESVs of woodland and water bodies did not change significantly. Only the ESV of grassland and unused land clearly surged.

**Figure 6:**
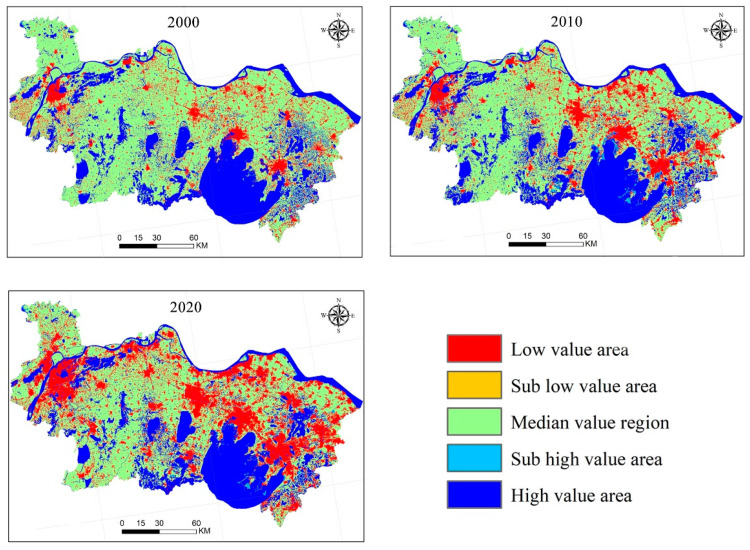
Spatial distribution of ecosystem service values in southern Jiangsu from 2000 to 2020.

The ESVs in southern Jiangsu from 2000 to 2020 were classified into five levels using the natural break approach in ArcGIS 10.5 (developed by ESRI in Redlands, CA, USA), and the same interval ranges were used in the 2000 and 2010 data to clearly illustrate changes. Figure 6 illustrates the spatial distribution of ESVs. Low-value areas are primarily urban built-up areas in southern Jiangsu; moreover, with the continuous expansion of urban built-up land, the spatial agglomeration of low-value areas is gradually becoming significant. High-value areas are primarily concentrated in the Taihu lake basin and the Yangtze river basin.

#### 3.2.3. Analysis of Changes in the Value of Individual Ecosystem Services

Values of each ecosystem service in southern Jiangsu can be acquired from the ESV coefficient and the land-use data from 2000 to 2020 (Table 5). In 2020, the individual ecosystem service values can be ranked as hydrological regulation > purifying the environment > climate regulation > gas conditioning > water supply biodiversity > food production > soil conservation > aesthetic landscape > raw material production > nutrient cycle maintenance. So, hydrological regulation is the most important ecosystem service in southern Jiangsu. The value of almost all ecosystem services dropped from 2000 to 2020, except water supply. The value of food production had the most significant decrease, which is connected with the decline in agricultural land.

### 3.3. The Coupling and Coordination between Tourism Economic Development and Ecosystem Service Value

The comprehensive evaluation indices of the tourism industry and environmental conditions in southern Jiangsu in 2000, 2010 and 2020—as well as the coupling coordination degree between them—were calculated using the aforementioned comprehensive evaluation model and coupling coordination model, as shown in Table 6. Due to the significant influence of the pandemic on tourism in 2020, this study uses the ESV in 2020 and the tourism economic development level in 2019. (1) The coupling degree and coupling coordination degree between tourism economy and ESV largely fluctuated. Both the coupling degree and coupling coordination degree surged from 2000 to 2010, then declined from 2010 to 2020, in southern Jiangsu. The tourism economy and ESV unstably coupled from 2000 to 2020. (2) Differences exist among the five southern Jiangsu cities. The coupling coordination degree surged in the beginning but then declined in Nanjing, Suzhou and Zhenjiang, while the degree gradually rose in Wuxi and stably increased in Changzhou.

## 4. Discussion

This paper aims to analyze the degree of coupling coordination between the tourism economy and ecosystem services in southern Jiangsu from 2000 to 2020 in order to provide the theoretical basis for coordinating the relationship between tourism development and ecological environment protection in southern Jiangsu. The development level of the tourism economy and ecosystem service level in southern Jiangsu were evaluated by a comprehensive evaluation model, the ESV was estimated and the land-use transition was analyzed. The coupling coordination degree between the two systems was then calculated using the coupling coordination degree model. Finally, the interaction mechanism of the two systems was elaborated. The main conclusions are as follows:

### 4.1. Comprehensive Evaluation of Subsystems

Firstly, the tourism development level in southern Jiangsu can be seen in Figure 3 and Figure 4. The comprehensive evaluation model is used to calculate the evaluation index system constructed in this study. The results are in accord with the actual situation of the regional ecological environment and tourism economic development, indicating that the index system has certain applicability [3,35]. The coupling coordination degree model is used to measure the coupling relationship between the ecosystem and the tourism economic system, which can provide guidance on methodologies for the coupling coordination analysis of the two systems and provide the basis for government decision-making [7,56].

Secondly, Table 2, Table 3, Table 4 and Table 5 and Figure 5 and Figure 6 reflect the land-use conversion and ESV in southern Jiangsu. The year 2010 can be regarded as a dividing line for the economic and social development of southern Jiangsu. This is because the implementation of the integration strategy of the Yangtze Delta in 2010 ensured a position of the Yangtze Delta region in the national strategy. Therefore, the tourism economy in southern Jiangsu also developed rapidly. In particular, Suzhou, which is close to Shanghai, has rapidly grown in its tourism economy, overtaking Nanjing, the capital of Jiangsu Province. The development level of the tourism economy in the five regions is related to geographical location, resource endowment, transportation infrastructure and other factors. The results of this study are consistent with related studies [7,19,41,57].

Thirdly, we can obtain the change in land-use type and the value of ecosystem services in southern Jiangsu. (1) There is a close relationship between the development of the tourism economy and land-use changes in southern Jiangsu. For example, water can provide a beautiful natural environment, fresh air and diversified flora and animal species, as well as play an important role in climate regulation, hydrological regulation and water supply. Wetland resources such as rivers and lakes can attract more tourists to come for leisure and recreation. Food production, raw material production, soil conservation, maintaining nutrient cycling and maintaining biodiversity are very important functions of cultivated land, which can also provide more agricultural leisure space and living security for tourists. In addition, historical and cultural heritage and other architectural landscapes can provide visitors with aesthetic appreciation, cultural entertainment and many other functions. Therefore, the ESV of diversified land use plays an important role in the tourism economy. (2) With the acceleration of urbanization in the Yangtze Delta, especially after 2010, the acquisition of a large amount of cultivated land and the expansion of built-up land has led to a downward trend in the value of ecosystem services. This is consistent with the previous related studies [26,27,58]. It can be inferred that the current development mode is not sustainable. The rapid development of the tourism economy has increased the tourist carrying capacity, but also led to a decline in ecological carrying capacity.

### 4.2. Analysis of the Coupling Coordination Relationship between the Tourism Economy and Ecosystem Service Functions

The coupling coordination degree between the tourism economy and ecosystem service functions is calculated with the coupling coordination model. The result shows an inverted U-shaped development regarding the coupling coordination degree between the two systems, and this result has a certain novelty. The tourism economy level in southern Jiangsu was relatively low in the early 21st century, and the impact of urban construction on ecosystem service functions was neglected; thus, the ESV remained at a low level. At this time, the tourism economy and ecosystem service functions restricted each other, and the coupling coordination degree between them was mild disorder at a low level. With the implement of the Yangtze Delta integration strategy and the publishing of the Yangtze River Delta Regional Plan, the tourism resources, the high-speed railway network and the flight network achieved abundance; so, the number of domestic and international tourists surged, and the tourism economy grew fast. Meanwhile, the Jiangsu government began to realize the impact of urbanization on ecosystem service value, and started the restoration of agricultural land, water bodies and woodland, which led to the coupling coordination degree between the two systems reaching intermediate coordination in 2010. However, while the tourism industry prospered and the number of tourists continued to grow, the contradiction between tourism development and ecological environment capacity emerged. Urban construction and expansion encroaches on ecological land, and the damage to the ecological service functions can hardly be compensated even if environmental protection is considered during development. The rapid growth of the tourism economy and decreasing ESV indicate the unsynchronized and uncoordinated development between the two systems; thus, the coupling coordination degree became mild disorder afterward. This inference is consistent with the discussions of previous studies [16,35,40,59]. In addition, there were spatial and temporal differences in the coupling coordination degree of the tourism economy and ESV among the five cities in southern Jiangsu, and it could be generated by a variety of factors such as economic development level, tourism resources and transportation infrastructure.

### 4.3. Coupling Mechanism of the Tourism Economy and Ecosystem Service Functions

**Figure 7:**
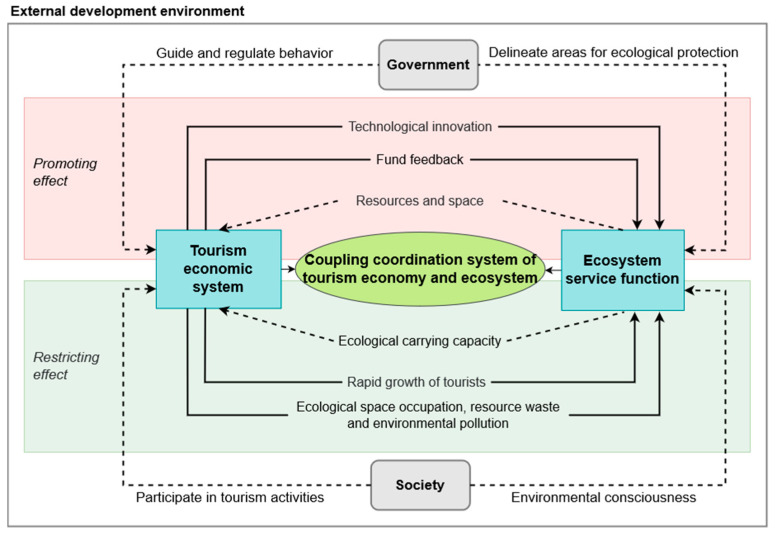
Coupling mechanism of tourism economic systems and ecosystem service function.

In this study, it can be found that the tourism economy and ecosystem promote and restrict each other while evolving together and promoting the sustainable development of the region. This section discusses the coupling mechanism of the tourism economic system and ecosystem in detail, expecting to provide a theoretical base for coordinating the relationship between them (Figure 7). Firstly, the ecosystem has both positive and negative effects on the tourism economy [54,60]. Abundant tourism resources and a beautiful natural and cultural environment are fundamental for the development of the tourism industry. A healthy ecological environment can attract tourists, and thus increase the economic benefits from tourism, while promoting tourism economical and regional development. Meanwhile, subsystems within the ecosystem constrain the development of a tourism economy, since the ecosystem capacity is limited. The development of a tourism economy may not exceed the capacity of ecosystem; otherwise, it will harm the ecological environment as well as the tourism economy. Secondly, the development of a tourism economy has both positive and negative effects on the ecosystem [1,61]. On one hand, the rapid development of a tourism economic system can financially support the restoration and protection of the ecosystem. With the development of the regional tourism industry, the benefits from tourism also increase, and it can provide necessary funding for ecosystem protection and restoration. On the other hand, the rapid growth of the tourism economy will put the local ecological environment under pressure. The increasing number of tourists and the spatial expansion of the tourism industry can impact the quality of subsystems such as water, soil and air, thus threatening the whole ecosystem. Moreover, overexploitation of resources and environmental pollution will affect the ecosystem service functions negatively. In addition, the coupling coordination structure interacts with many stakeholders including the government, society and the external environment [62].

## 5. Conclusions

In this study, a comprehensive evaluation model and coupling coordination degree model were adopted to measure the development level of the tourism economy, ecosystem service value and the coupling coordination degree between the tourism economy and ecosystem service value in southern Jiangsu from 2000 to 2020. The following are the main conclusions: (1) From 2000 to 2020, the development of the tourism economy in southern Jiangsu showed periodic fluctuations; especially after 2010, with the proposed integration strategy of the Yangtze Delta, the tourism economy in southern Jiangsu developed rapidly. Due to the variance of urban resources, location, investment conditions, etc., the development of the tourism economy shows spatial and temporal differences between cities. (2) As an important tourist destination, with the rapid development of the tourism economy and the expansion of urban built-up areas, the value of various ecosystem services in southern Jiangsu has been declining. In addition, there are differences in the value of ecosystem services among different land-use types. (3) The coupling coordination degree of the tourism economy and ecosystem service value in southern Jiangsu Province presents an inverted U-shape, and the research results have certain novelty. At the same time, the coupling coordination degrees of the five cities in southern Jiangsu are also different. Lastly, this study reveals the interaction mechanism in the coupling coordinated system of the tourism economy and ecosystem service functions. Suggestions are provided regarding the sustainable development of the coupling coordinated system in the region, which can be a reference for tourism and regional development in southern Jiangsu and even the whole Yangtze Delta region.

Based on the results above, the following policy implications can be suggested. Although the growth of tourism opens up new possibilities for urban development, its effects on ecosystem services must also be taken into account [63]. First, the government needs to make strict ecological protection policies and regulations, put strict restrictions on tourism land, make ecological control lines to protect water bodies, forest lands, grasslands and agricultural land and find the balance between ecological protection and human well-being. Land-use planning fully considers supply services, regulation services, support services and cultural services. Second, ecotourism should be actively promoted by the tourism industry. A win–win situation of ecological protection and tourism revenue can be achieved through the growth of ecotourism, which can also address the growing needs of tourists in terms of ecology, health and education. To minimize the negative effects of tourism on the ecological environment, technical tools can be used for environmental monitoring and tourism-flow directing. Third, visitors need to be responsible for the environment, raise their awareness of ecological protection, develop eco-friendly consumption habits and adopt an eco-friendly lifestyle.

This study uses a variety of research methods to examine the coupling coordination between the ecosystem service value and the tourism economy. The research methods are practical, can reflect the actual development of the region and can be used as a reference for regional industrial structure upgrading, preservation of the natural environment, policy-making, etc. However, the coupling coordinated system of ecosystem services and tourism economies is a complex system [25,33], and this study only briefly reveals the coupling coordination between these two due to space limitations. More detailed interaction mechanisms and the paths between the tourism economy and ecological service functions will be further explored in the future.

## Figures and Tables

**Table 1:** The index system of tourism economic development level in southern Jiangsu.

Level-1 Indicator	Level-2 Indicator	Unit	Attribute
Tourism facilities (X_1_)	Number of scenic spots (X_11_)	_	+
	Number of travel agencies (X_12_)	_	+
	Number of star-grade hotels (X_13_)	_	+
Tourism revenue (X_2_)	Income from domestic tourism (X_21_)	yuan	+
	Foreign exchange earnings from tourism (X_22_)	yuan	+
Tourism scale (X_3_)	Number of domestic tourists received (X_31_)	person	+
	Number of overseas tourists received (X_32_)	person	+
Tourism employment (X_4_)	Employment in accommodation and catering (X_41_)	person	+
	Employment in culture, sports and entertainment (X_42_)	person	+

**Table 2:** Land-use area in southern Jiangsu from 2000 to 2020 [Unit: hm^2^] and its proportion [Unit: %].

Particular Year	Index	Agricultural Land	Woodland	Grassland	Water Body	Built-Up Land	Unused Land
2000	Area (hm^2^)	1,771,683.03	189,418.10	4443.48	574,478.00	269,796.10	15.03
	Proportion (%)	63.0530	6.7413	0.1581	20.4453	9.6019	0.0005
2010	Area (hm^2^)	1,599,376.23	177,901.40	24,303.69	604,371.70	403,861.10	19.44
	Proportion (%)	56.9207	6.3314	0.8650	21.5092	14.3731	0.0007
2020	Area (hm^2^)	1,362,938.13	191,166.80	20,035.44	572,845.70	662,639.60	208.35
	Proportion (%)	48.5060	6.8035	0.7130	20.3872	23.5829	0.0074
2000–2010	Variation (hm^2^)	−172,306.80	−11,516.70	19,860.21	29,893.68	13,4065.10	4.41
	Dynamic degree (%)	−0.9726	−0.6080	44.6952	0.5204	4.9691	2.9341
2010–2020	Variation (hm^2^)	−236,438.10	13,265.46	−4268.25	−31,526.00	258,778.40	188.91
	Dynamic degree (%)	−1.4783	0.7457	−1.7562	−0.5216	6.4076	97.1759
2000–2020	Variation (hm^2^)	−408,744.90	1748.79	15,591.96	−1632.33	392,843.50	193.32
	Dynamic degree (%)	−1.1535	0.0462	17.5448	−0.0142	7.2804	64.3114

**Table 3:** Land-use conversion matrix from 2000 to 2020 (hm^2^).

2000	2020						Roll-Out Volume
	Grassland	Agricultural Land	Built-Up Land	Woodland	Water Body	Unused Land	
Grassland	1927.53	261.00	550.62	1139.13	556.92	8.28	2515.95
Farmland	7138.62	1,253,738.52	396,801.27	35,378.28	78,541.38	84.24	517,943.79
Built-up land	1207.44	31,860.63	228,799.80	2915.19	5007.42	5.58	40,996.26
Woodland	6611.04	16,132.77	11,591.19	150,629.31	4371.03	82.71	38,788.74
Water body	3146.76	60,944.04	24,896.52	1103.94	484,363.80	18.90	90,110.16
Unused land	4.05	1.17	0.18	0.99	0.00	8.64	6.39
Roll-in volume	18,107.91	109,199.61	433,839.78	40,537.53	88,476.75	199.71	

**Table 4:** Total ESV in southern Jiangsu [unit: CNY 100 million] and the changes [unit: %].

Year	Agricultural Land	Woodland	Grassland	Water Body	Unused Land
2000	699.8148	415.3938	2.4395	7216.0180	0.0003
2010	631.7536	390.1377	13.3427	7591.5130	0.0004
2020	538.3606	419.2289	10.9995	7195.5150	0.0042
2000–2010	−0.97%	−0.61%	44.70%	0.52%	2.93%
2010–2020	−1.48%	0.75%	−1.76%	−0.52%	97.18%
2000–2020	−1.15%	0.05%	17.54%	−0.01%	64.31%

**Table 5:** Value of individual ecosystem services in southern Jiangsu from 2000 to 2020 [unit: CNY 100 million] and proportion of change [unit: %].

Ecosystem Service Functions		2000	2010	2020	2000–2010	2010–2020	2000–2020
Supply services	Food production	260.559	242.503	212.419	−0.69%	−1.24%	−0.92%
	Raw material production	61.025	57.816	53.161	−0.53%	−0.81%	−0.64%
	Water supply	179.765	233.798	248.507	3.01%	0.63%	1.91%
Regulate the service	Gas conditioning	255.475	239.840	217.346	−0.61%	−0.94%	−0.75%
	Climate regulation	343.148	336.818	325.728	−0.18%	−0.33%	−0.25%
	Purifying the environment	381.045	393.851	375.085	0.34%	−0.48%	−0.08%
	Hydrological regulation	6278.923	6549.959	6188.132	0.43%	−0.55%	−0.07%
Support services	soil conservation	169.093	163.800	155.323	−0.31%	−0.52%	−0.41%
	Nutrient cycle maintenance	37.951	35.100	31.103	−0.75%	−1.14%	−0.90%
	Biodiversity	223.924	226.905	217.520	0.13%	−0.41%	−0.14%
Cultural services	Aesthetic landscape	142.759	146.357	139.785	0.25%	−0.45%	−0.10%

**Table 6:** Coupling and coordination degree of tourism economy and ecosystem service value in southern Jiangsu.

Region	Year	Coupling Degree C Value	Coordination Index T Value	Coupling Compatibility D Value	Coupling Coordination Degree
Southern Jiangsu region	2000	0.215	0.429	0.303	Mild disorder
	2010	0.826	0.633	0.724	Intermediate coordination
	2020	0.199	0.500	0.315	Mild disorder
Nanjing	2000	0.763	0.403	0.554	Grudging coordination
	2010	0.948	0.751	0.843	Good coordination
	2020	0.215	0.429	0.303	Mild disorder
Wuxi	2000	0.824	0.303	0.500	On the verge of disorder
	2010	1.000	0.497	0.705	Intermediate coordination
	2020	0.981	0.742	0.853	Good coordination
Changzhou	2000	0.716	0.241	0.415	On the verge of disorder
	2010	0.798	0.549	0.662	Primary coordination
	2020	0.881	0.487	0.655	Primary coordination
Suzhou	2000	0.586	0.538	0.562	Grudging coordination
	2010	0.995	0.456	0.673	Primary coordination
	2020	0.429	0.520	0.472	On the verge of disorder
Zhenjiang	2000	0.216	0.422	0.302	Mild disorder
	2010	1.000	0.501	0.708	Intermediate coordination
	2020	0.647	0.302	0.442	On the verge of disorder

## Data Availability

The original contributions presented in the study are included in the article; further inquiries can be directed to the corresponding author.

## Appendix A

**Table A1:** South Jiangsu Ecosystem Service Value Coefficient [Unit: 10,000 yuan/(hm^2^·year)].

Ecosystem Service Function		Farmland	Woodland	Grassland	Waters	Unused Land
Supply services	Food production	1.18	0.29	0.11	0.80	0
	Production of material	0.20	0.65	0.15	0.23	0
	Water supply	−1.71	0.34	0.09	8.29	0
Regulatory services	Gas conditioning	0.96	2.16	0.55	0.77	0.02
	Climate regulation	0.50	6.46	1.45	2.29	0
	Clean the situation	0.15	1.87	0.48	5.55	0.10
	Hydrological regulation	1.87	3.89	1.06	102.24	0.03
Support services	Soil conservation	0.37	2.63	0.67	0.93	0.02
	Maintain nutrient cycle	0.17	0.20	0.05	0.07	0
	Bio-diversity	0.18	2.39	0.61	2.55	0.02
Cultural services	Aesthetic landscape	0.08	1.05	0.27	1.89	0.01

Note: This study sets the ecosystem service value of built-up land (mainly hard impermeable layer, excluding water area, green land, etc.) as 0.

**Table A2:** Classification of coupling coordination degree.

Coupling Coordination Degree (D)	Coordination Level	Coupling Coordination Degree (D)	Coordination Level
0.00~0.09	Extreme disorder	0.50~0.59	Grudging coordination
0.10~0.19	Severe disorder	0.60~0.69	Primary coordination
0.20~0.29	Moderate disorder	0.70~0.79	Intermediate coordination
0.30~0.39	Mild disorder	0.80~0.89	Good coordination
0.40~0.49	On the verge of disorder	0.90~1.00	Quality coordination
